# Supplementary material for: A real-world disproportionality analysis of Tenofovir Alafenamide (TAF): Data mining of the FDA adverse event reporting system (FAERS)
Source: PLoS One. 2025 Jun 5;20(6):e0324675. doi: 10.1371/journal.pone.0324675 (PMC12140211; doi:10.1371/journal.pone.0324675)
Supplement: Attachment — Submitted filename: Response to Reviewers.docx [file pone.0324675.s002.docx]

Response: Thank you very much to the reviewer for pointing out the

shortcomings to us.

The review comments of the reviewers have been replied to item by item in detail,

and the content of the article has been revised strictly in accordance with the

requirements of the reviewers.

*academic editor*

**1.Please also emphasize throughout the Discussion when the adverse effects that were associated with use of the medicaion in your analysis were not previosuly detetcted in clinical studies of the medication, as they might not be causally related and thus may not be clinical signifcance. This is currently mentioned in the Limitations section, but it is important to mention in the body of the Discussion prior to this as well.**

Reinforced Explanation for the Discussion Section
We have added three important paragraphs in the main body of the discussion (Lines 290 - 295, 325 - 338, and 330 - 333). These revisions make the clinical interpretation of risk signals more scientific.

**Modify lines 295 - 300 on page 14.**While IRIS is a recognized complication of antiretroviral therapy in HIV, its association with TAF in HBV monotherapy is unexpected and may reflect off-label use in HIV/HBV co-infected populations or misclassification of immune flare events. This signal underscores the importance of contextualizing pharmacovigilance findings within prescribing patterns and patient heterogeneity.

**Modify lines 330 - 333 on page 15.**However, as these associations were absent in pre-approval trials, they may reflect residual confounding rather than direct causation. Clinicians should interpret these signals cautiously, balancing potential risks against established benefits.

**Modify lines 335 - 338 on page 15.**While this finding could indicate emerging resistance patterns in real-world use, it may also result from misreported virologic breakthrough due to non-adherence rather than true resistance. This highlights the need for confirmatory studies to assess causality.

**2.Please ensure that your manuscript meets PLOS ONE's style requirements, including those for file naming.**

The full text has been adjusted according to the latest format requirements of PLOS ONE.

**3.PLOS requires an ORCID iD for the corresponding author in Editorial Manager on papers submitted after December 6th, 2016. Please ensure that you have an ORCID iD and that it is validated in Editorial Manager.**

The ORCID iD of the corresponding author has been verified in the system.

**4.We note that your Data Availability Statement is currently as follows: [All relevant data are within the manuscript and its Supporting Information files.]**

The Data Availability Statement has been revised as follows:This study was performed using the FAERS source that was provided by the FDA. The database used in this study is publicly available in Website of

[https://www.fda.gov/drugs/fdas-adverse-event-reporting-system-faers/fda-adverse-event-reporting-system-faers-latest-quarterly-data-files](https://www.fda.gov/drugs/fdas-adverse-event-reporting-system-faers/fda-adverse-event-reporting-system-faers-latest-quarterly-data-files)

**5. Please amend either the abstract on the online submission form (via Edit Submission) or the abstract in the manuscript so that they are identical.**

The abstract content in the online submission system and that in the manuscript have been double-checked in both directions to ensure they are completely identical.

Reviewer #1:

**1.Introduction: Includes a lot of run-on sentences and some grammatical errors. Authors should consider subheadings to break up sections to help the reader, as well as use a grammar checking software to restructure some awkward sentences.**

**Modify on page 2-4.**

The sub - titles have been set as required by the reviewers, and the content has been revised. A new third - level sub - title has been added:①Hepatocellular Carcinoma and Hepatitis B

②Antiviral Therapy

③The FDA Adverse Event Reporting System (FAERS)

④Tenofovir Alafenamide

2. **Methods: Please describe which suite of tools were used to execute which methodologies. Also, were you comparing methodologies? Maybe provide a citation as to why those four analytic methodologies were chosen.**

I've added a new paragraph to explain the selection criteria for the four signal detection methods. What has been added is:

**Modify lines 182 - 194 on page 5-6.**

Multiple lines of evidence indicate that the Reporting Odds Ratio (ROR) and the Proportional Reporting Ratio (PRR) are highly sensitive in detecting safety signals, especially suitable for the rapid screening of high-frequency events. The Bayesian Confidence Propagation Neural Network (BCPNN) method performs excellently in handling small samples and rare events. Although its sensitivity is lower than that of PRR and ROR, it has better specificity and provides relatively more stable signals. It can provide more accurate signal detection in specific scenarios. The Multi-item Gamma Poisson Shrinker (MGPS) method has significantly higher specificity than other methods. The MGPS method is particularly suitable for scenarios that require high specificity, such as reducing the misdiagnosis rate [33]. The comprehensive use of multiple methods (such as PRR, ROR, BCPNN, and MGPS) can significantly improve the reliability of signal detection.

**3.Typos:**

**-Line 154 indicates a different time period of study (Q1 2001 - Q1 2023) than lines 145-146 (4th quarter of 2016 to 1st quarter of 2023).**

**Modify lines 205 on page 6.**

The research period has been unified as:(4th quarter of 2016 to 1st quarter of 2023).

**-Table 1. Please review as there are some symbol errors/typos. Also, please type out the full meaning of a word before using the abbreviations in the text and in the footnotes of the table.**

**Modify on page 6.**

The spelling errors and textual mistakes in the table have been corrected. The full meanings of all abbreviations have been supplemented. For instance: United States (US)，Japan (JP)，China (CN)，Turkey (TR)。

Reviewer #2:

**Why are you using the FAERS database to study this drug, it should be that someone else has done this with this similar database similar methodology, so you can write cite in the INTRODUCTION section about some specific other similar studies, such as recommending a few (It is equivalent to saying that someone else has done this type of research using the FAERS database, and you can use this database to do research related to TAF as well**

### I've described the reasons for using the FAERS database for analysis. Key literature citations have been added to the third part of the introduction. These supplements fully demonstrate the reliability and innovation of the methodology.

**Modify lines 71-97 on page 3.**

### The FDA Adverse Event Reporting System (FAERS) has been widely validated as a critical tool for post-marketing pharmacovigilance, particularly in detecting rare or delayed adverse drug reactions (ADRs) that may escape detection in clinical trials. Recent pharmacoepidemiologic studies have demonstrated the utility of disproportionality analysis on FAERS data across diverse therapeutic domains. For instance, Wang et al. ^[14]^ identified novel cardiovascular risks associated with sildenafil through ROR-based signal detection, revealing dose-dependent QT prolongation signals (ROR=3.41, 95% CI 2.89-4.02) not fully characterized in pre-approval studies. Similarly, Zhao et al. ^[15]^ applied Bayesian confidence propagation neural network (BCPNN) methods to uncover everolimus-associated interstitial lung disease signals (IC025=2.7), demonstrating FAERS' capacity to detect immune-mediated toxicities. In oncology drug safety, Yang et al. ^[16]^ leveraged FAERS to quantify topotecan-related myelosuppression risks, establishing significant associations with thrombocytopenia (PRR=4.12, χ²=356.8) that aligned with real-world clinical observations.The system's analytical power extends to both inter-class comparisons and intra-class risk stratification. Zhao et al. ^[17]^ revealed acetylsalicylic acid's elevated gastrointestinal bleeding risk in elderly populations (ROR=5.24, PRR=4.87), corroborating clinical findings on age-dependent mucosal vulnerability. In antineoplastic drug surveillance, vinorelbine demonstrated significantly higher neutropenia risk than vincristine (PRR=6.15 vs. 3.92), a differential risk profiling methodologically consistent with adjusted ROR (aROR) strategies in thrombolytic agent studies, where alteplase showed 88% higher angioedema risk than tenecteplase ^[18]^. These cases collectively validate FAERS' dual capability in detecting cross-class safety signals and refining risk gradients within drug classes through standardized disproportionality metrics (ROR/PRR) and confounder-adjusted methodologies.

[15] Zhao B, Fu Y, Cui S, Chen X, Liu S, Luo L. A real-world disproportionality analysis of Everolimus: data mining of the public version of FDA adverse event reporting system. Front Pharmacol. 2024;15:1333662. Published 2024 Mar 12. doi:10.3389/fphar.2024.1333662

[16] Yang H, Wan Z, Chen M, Zhang X, Cui W, Zhao B. A real-world data analysis of topotecan in the FDA Adverse Event Reporting System (FAERS) database. Expert Opin Drug Metab Toxicol. 2023;19(4):217-223. doi:10.1080/17425255.2023.2219390

[17] Zhao B, Zhang X, Chen M, Wang Y. A real-world data analysis of acetylsalicylic acid in FDA Adverse Event Reporting System (FAERS) database. Expert Opin Drug Metab Toxicol. 2023;19(6):381-387. doi:10.1080/17425255.2023.2235267

[18] Zhong C, Zheng Q, Zhao B, Ren T. A real-world pharmacovigilance study using disproportionality analysis of United States Food and Drug Administration Adverse Event Reporting System events for vinca alkaloids: comparing vinorelbine and Vincristine. Expert Opin Drug Saf. 2024;23(11):1427-1437. doi:10.1080/14740338.2024.2410436

**1.Clarity and Grammar: Improve sentence structures and grammar throughout the manuscript to enhance readability. For example, in the **Abstract**, the sentence *"TAF is a new class of drugs approved for the treatment of viral hepatitis B."* could be revised to *"TAF is a novel antiviral drug approved for the treatment of hepatitis B virus (HBV) infection."***

The grammar and structure of the Abstract section have been revised.

**Modify lines 10-13 on page 1.**

**2.Abstract Improvements: The **Abstract** should briefly mention key findings, including unexpected adverse events. The conclusion should explicitly state the significance of these findings for clinical practice.**

**Modify lines 29-39 on page 1.**

These findings unearth novel neurological, metabolic, and resistance - related risks, thereby necessitating a marked increase in clinical vigilance. The identification of signals related to cerebral infarction and dementia implies potential vascular/metabolic interplay, highlighting the importance of lipid monitoring among long - term tenofovir alafenamide (TAF) users. In response, healthcare providers should prioritize strengthening the monitoring of neurological symptoms and lipid profiles, reevaluating bone health assessment and management protocols especially in high - risk populations, and providing support to enhance patient adherence to mitigate resistance risks. This analysis offers crucial post - marketing evidence, which is instrumental in optimizing the risk - benefit balance of TAF in the long - term management of chronic hepatitis B virus (HBV) infection.

**3.Methodology Details: Provide a clearer explanation of statistical methodologies such as **BCPNN** and **GPS**, ensuring non-specialist readers understand their application.**

The description of statistical methods has been supplemented in the "Statistical analysis" section.

**Modify lines 155 - 194 on page 5-6.**

①ROR method：Detect the preliminary association signals between drugs and adverse events

②PRR method：Evaluate the degree of abnormality of the reporting frequency of drug evaluation reports relative to the background population.

③BPCNN method：Reduce false positives caused by the fluctuations of small-sample data through Bayesian shrinkage.

④EBGM method：Handle large-scale sparse datasets for robust signal detection

Multiple lines of evidence indicate that the Reporting Odds Ratio (ROR) and the Proportional Reporting Ratio (PRR) are highly sensitive in detecting safety signals, especially suitable for the rapid screening of high-frequency events. The Bayesian Confidence Propagation Neural Network (BCPNN) method performs excellently in handling small samples and rare events. Although its sensitivity is lower than that of PRR and ROR, it has better specificity and provides relatively more stable signals. It can provide more accurate signal detection in specific scenarios. The Multi-item Gamma Poisson Shrinker (MGPS) method has significantly higher specificity than other methods. The MGPS method is particularly suitable for scenarios that require high specificity, such as reducing the misdiagnosis rate [33]. The comprehensive use of multiple methods (such as PRR, ROR, BCPNN, and MGPS) can significantly improve the reliability of signal detection.

**4.Data Presentation: Tables should be formatted for consistency. For example:**

**- Align column headers for better readability.**

**- Ensure numerical values have consistent decimal places.**

**- Clearly define abbreviations (e.g., **SOC, PT**).**

**Modify on page 7-13.**

The format of the table has been modified, and the data has been unified to a consistent number of decimal places. The definitions of SOC (System Organ Class) and PT (Preferred Term) have been clarified.

5. **Discussion Enhancement:**

**- Clarify the implications of **unexpected adverse events** such as **cerebral infarction and dementia**. Are these findings clinically significant?**

**- Provide possible biological explanations or cite relevant literature.**

**- Discuss potential confounding factors.**

I have analyzed cerebral infarction and dementia among the adverse reactions and added a paragraph:

**Modify lines 311 - 333 on page 15.**

Cerebral infarction and dementia have not been reported in previous studies, it is noteworthy that recent studies suggest TAF's impact on lipid metabolism may indirectly elevate the risk of neurological events. A new study demonstrated that in chronic hepatitis B (CHB) patients switching from tenofovir disoproxil fumarate (TDF) to TAF, significant increases in low-density lipoprotein cholesterol (LDL-C) and triglycerides (TG) were observed, accompanied by reduced high-density lipoprotein cholesterol (HDL-C) ^[30]^. Five-year follow-up data from two phase 3 clinical trials further confirmed that prolonged TAF use leads to a progressive rise in LDL-C and TG levels, alongside a sustained decline in HDL-C^[31]^. Epidemiological evidence indicates that persistently elevated LDL-C may heighten dementia risk through mechanisms such as promoting cerebral atherosclerosis and β-amyloid deposition. Additionally, TAF-associated dyslipidemia may synergize with other risk factors (e.g., hypertension, diabetes) to accelerate atherosclerosis, thereby increasing the incidence of cerebrovascular events such as cerebral infarction^[32]^. These findings highlight that TAF's metabolic effects should be a critical consideration in overall safety evaluations, particularly for patients with preexisting cardiovascular risk factors or baseline lipid abnormalities. In clinical practice, enhanced lipid monitoring (every 3-6 months) and tailored interventions are warranted, including statin therapy or switching to antiviral medications with lesser lipid impact. However, as these associations were absent in pre-approval trials, they may reflect residual confounding rather than direct causation. Clinicians should interpret these signals cautiously, balancing potential risks against established benefits.

**6. Reference Formatting: Ensure all references are correctly cited in **PLOS ONE format** and include DOI links where applicable.**

The format of the references has been standardized.

7. **Figure and Table Legends: Each table/figure should have **self-explanatory captions**, detailing key insights for readers.**

The format of the references has been standardized.

**Modify lines 233-236 on page 9.**

**Modify lines 258-259 on page 14.**

**8. Ethical Considerations: If patient data were used, confirm compliance with ethical guidelines and data privacy regulations.**

The data are derived from the FAERS database, and ethical approval is not required.

8. **Can Author put a bar chart for the percentage of TAF uses in the top five country and hospitalization, death, life threating events, and disability.**

In Table 1, the following has been added:

**Modify on page 7.**

Hospitalization (11.14%) was the most frequently reported severe outcome, followed by death and life-threatening occurring in 99 (5.18%) and 54 (2.82%) cases, respectively. The incidence of disability was the lowest (1.10%).

We believe these revisions comprehensively address all concerns. Please don't hesitate to contact us for any additional modifications.

Sincerely,
Li
