# Supplementary material for: A real-world disproportionality analysis of Tenofovir Alafenamide (TAF): Data mining of the FDA adverse event reporting system (FAERS)
Source: PLoS One. 2025 Jun 5;20(6):e0324675. doi: 10.1371/journal.pone.0324675 (PMC12140211; doi:10.1371/journal.pone.0324675)
Supplement: Attachment — Submitted filename: Response_to_Reviewers_auresp_2.docx [file pone.0324675.s003.docx]

Response: Thank you very much to the reviewer for pointing out the

shortcomings to us.

The review comments of the reviewers have been replied to item by item in detail,

and the content of the article has been revised strictly in accordance with the

requirements of the reviewers.

****Comment 1:**Please write out all abbreviations at first mention, including in the Abstract and the main text of the paper.**

****Response:****Thank you for your careful reading. We have revised the manuscript to ensure all abbreviations are spelled out at first mention. Specifically:

**Modify lines 11-15 on page 1.**

**Modify lines 62-63 on page 3.**

****Comment 2:**The Introduction contains many statements that must be supported by references. For example, the section on TAF refers to many aspects of TAF's safety record without references that are needed. Please review the entire manuscript and add references to support statements based on prior studies and literature.**

****Response:**We appreciate this constructive suggestion. We have added supporting references to strengthen the statements about TAF's safety and efficacy:**

**Modify lines 104-118 on page 4**

**We have strengthened the discussion of TAF's therapeutic benefits in the Introduction (lines 104-109, page 4) by incorporating supporting evidence from Lim et al ^[34]^.**

**In lines 109-114 of page 4, the treatise by Terrault NA et al. has been added to demonstrate the advantages of Tenofovir Alafenamide (TAF) in the protection of the kidneys and bones ^[13]^.**

**In lines 114-118 of page 4, the treatise by Agarwal K et al. has been incorporated to demonstrate that Tenofovir Alafenamide (TAF) has a relatively long plasma half-life and requires a lower dosage^[35]^.**

**[13]Terrault NA, Lok ASF, McMahon BJ, et al. Update on prevention, diagnosis, and treatment of chronic hepatitis B: AASLD 2018 hepatitis B guidance. Hepatology. 2018;67(4):1560-1599. doi:10.1002/hep.29800**

**[34]Lim YS, Chan HLY, Ahn SH, et al. Tenofovir alafenamide and tenofovir disoproxil fumarate reduce incidence of hepatocellular carcinoma in patients with chronic hepatitis B. JHEP Rep. 2023;5(10):100847. Published 2023 Jul 13. doi:10.1016/j.jhepr.2023.100847**

**[35]Agarwal K, Brunetto M, Seto WK, et al. 96 weeks treatment of tenofovir alafenamide vs. tenofovir disoproxil fumarate for hepatitis B virus infection. J Hepatol. 2018;68(4):672-681. doi:10.1016/j.jhep.2017.11.039**

****Comment 3. Please capitalize the first word in each row of the tables (e.g., Sex).****

****Response:****

**Modify on page 7**

**I have made the necessary revisions to the tables on page 7. In accordance with the requirement, the first word of each row in the tables has been capitalized.**

****Comment 4:In Table 2, please define what is meant be "Investigations" as a category.****

****Response:****

**Modify lines 240-242 on page 10**

**Add the following supplementary explanation for "Investigations":**

*" Investigations" refers to diagnostic tests, laboratory findings, or monitoring procedures (e.g., blood tests, imaging studies, liver function assessments) as defined by the MedDRA® classification system.*

****Comment 5. In Table 3, the authors examined liver tests in multiple ways (ALT, AST, transaminases, etc.). Please provide a justification for this or else present analyses that include all of these as a single category of abnormal transaminases.****

****Response:****

**We have added an explanation detailing the rationale behind the separate analyses of the four aspects of liver tests: "ALANINE AMINOTRANSFERASE INCREASED," "LIVER FUNCTION TEST INCREASED," "TRANSAMINASES INCREASED," and "HEPATIC FUNCTION ABNORMAL.**

**Modify lines 310-330 on page 15**

The analysis identified multiple hepatic-related Preferred Terms (PTs) associated with TAF, including Alanine aminotransferase increased (ROR=4.02, PRR=6.07), Transaminases increased (ROR=4.73, PRR=8.53), and Hepatic function abnormal (ROR=2.70, PRR=5.01). While these PTs are distinct within the MedDRA® hierarchy (e.g., Alanine aminotransferase increased belongs to Investigations, whereas Hepatic function abnormal is categorized under Hepatobiliary disorders), they collectively align with the hepatotoxicity profile described in TAF's prescribing information, which explicitly lists ALT elevation as an adverse reaction. The signal for Transaminases increased (a broader category encompassing ALT/AST) and Hepatic function abnormal (reflecting systemic dysfunction) may represent varying clinical manifestations or reporting granularity of the same underlying hepatic injury mechanism. Importantly, these findings do not suggest novel liver toxicity patterns but rather reinforce the need for vigilant monitoring of liver function across multiple biomarkers (including transaminases, synthetic function tests, and clinical assessments), as recommended in current guidelines. Maintaining separate PTs in Table 3 adheres to MedDRA® pharmacovigilance standards while allowing clinicians to recognize both specific biochemical abnormalities and broader hepatic dysfunction signals. When taking this drug, it is more likely that alanine aminotransferase, transaminase and abnormal liver function will appear. Therefore, it is important to check liver function indicators regularly and, if necessary, use liver protective drugs or other antiviral drugs for treatment.

****Comment 6 . In line 300, the authors mention TAF being used off-label for HIV or hepatitis B, but this medication seems to have FDA approval for both infections. Please correct or clarify and justify.****

****Response:****

**In accordance with the U.S. Food and Drug Administration (FDA) prescribing guidelines, tenofovir alafenamide fumarate (TAF) should not be used as monotherapy for HIV-1 infection in patients co-infected with hepatitis B virus (HBV) and HIV-1, due to the risk of inducing HIV-1 resistance mutations. For this population, TAF must be administered in fixed-dose combinations with other antiretroviral agents, such as emtricitabine/tenofovir alafenamide fumarate (F/TAF), emtricitabine/rilpivirine/tenofovir alafenamide fumarate (FTC/RPV/TAF), and similar combination formulations. Importantly, our analysis specifically focused on TAF monotherapy for HBV infection, which necessitated revisions to lines 301-306 on page 15 of the manuscript. These modifications clarify the scope of our investigation and prevent potential misinterpretations regarding clinical applications：**

**Modify lines 301-306 on page 15**

The unexpected signal for immune reconstitution inflammatory syndrome (IRIS) (n=21, ROR=36.79) may reflect residual confounding from misclassified HBV flare events or undocumented HIV co-infection in FAERS reports. However, our analysis strictly excluded TAF use in HIV contexts, as monotherapy for HIV is contraindicated due to resistance risks. This underscores the importance of adhering to TAF’s approved indication for HBV monotherapy.
